# Influence of sociodemographic and obstetric factors on maternal mortality in Brazil from 2011 to 2021

**DOI:** 10.1186/s12905-024-02925-3

**Published:** 2024-02-01

**Authors:** Eric Renato Lima Figueiredo, Claudia do Socorro Carvalho Miranda, Ana Cristina Viana Campos, Fabiana de Campos Gomes, Cibele Nazaré Câmara Rodrigues, João Simão de Melo-Neto

**Affiliations:** 1 Urogenital System Clinical and Experimental Research Unit, Institute of Health Sciences, Federal University of Pará (UFPA), Belém, PA 66075-110 Brazil; 2 Laboratory of Epidemiology and Geoprocessing of Amazon, State University of Pará (UEPA), Belém, PA 66113-010 Brazil; 3 Laboratory and Observatory in Surveillance and Social Epidemiology, Federal University of the South and Southeast of Pará (Unifesspa), Marabá, PA 68500-000 Brazil; 4 Ceres Medical School (FACERES), São José Do Rio, Preto, PA 15090-305 Brazil

**Keywords:** Maternal mortality, Health inequalities, Public health surveillance, Women's health, Maternal and Child Health

## Abstract

**Background:**

Obstetric causes are classified as direct (complications of pregnancy, childbirth or the puerperium) or indirect (caused by pregnancy but not directly caused by it). This study aimed to analyze maternal mortality from obstetric causes in Brazil from 2011 to 2021.

**Methods:**

This was an ecological study on mortality and live births. The outcomes were the specific risk of mortality from direct and indirect cause adjustment and death during pregnancy and the puerperium. Binary and multiple linear logistic regressions were used to assess the influence of sociodemographic factors and maternal and child health indicators on maternal mortality and time of death (pregnancy and puerperium).

**Results:**

Regarding mortality during pregnancy and during the puerperium, increased (*p* = 0.003) and decreased (*p* = 0.004) mortality over the years, respectively; residing in the northern region was associated with lower (*p* < 0.05) and greater (*p* = 0.035) odds; and the Maternal Mortality Committee was the primary and least active source of investigation, respectively (*p* < 0.0001). The number of deaths from indirect causes increased with age (*p* < 0.001) and in the northern region (*p* = 0.011) and decreased in the white (< 0.05) and stable union (0.002) regions. Specifically, for mortality risk, the age group [women aged 15–19 years presented an increase in cesarean section (*p* < 0.001) was greater than that of women who had < 4 antenatal visits (*p* < 0.001)], education [women who completed high school (8 to 11 years) was greater when they had < 4 prenatal visits (*p* = 0.018)], and marital status [unmarried women had more than 4 antenatal visits (*p* < 0.001); cesarean birth (*p* = 0.010) and < 4 antenatal visits (*p* = 0.009) were predictors of marriage; and women in a stable union who had < 4 prenatal visits and live births to teenage mothers (*p* < 0.001) were predictors]. Women who had no education (*p* = 0.003), were divorced (*p* = 0.036), had cesarean deliveries (*p* < 0.012), or lived in the north or northeast (*p* < 0.008) had higher indirect specific mortality risk.

**Conclusions:**

Sociodemographic factors and maternal and child health indicators were related to different patterns of obstetric mortality. Obstetric mortality varied by region, marital status, race, delivery, prenatal care, and cause of death.

**Supplementary Information:**

The online version contains supplementary material available at 10.1186/s12905-024-02925-3.

## Background

Maternal mortality, defined as death during pregnancy or within 42 days of childbirth, is a global public health problem linked to socioeconomic disparities, the availability of health care, and inadequate obstetric care [1]. The maternal mortality indicator is crucial for guiding maternal health policies, especially those related to the quality of obstetric care [2]. Obstetric causes, which are classified as direct or indirect, contribute significantly to maternal deaths [3]. Direct obstetric maternal mortality occurs during pregnancy, childbirth, or the postpartum period as a result of obstetric complications. Indirect causes of pregnancy can include preexisting pathologies or pathologies that develop during pregnancy and are not directly caused by obstetric factors but are exacerbated by physiological changes [4].

Reducing maternal mortality remains a global challenge, as outlined in the 2000–2015 UN agenda (MDGs) and continued in the 2030 Sustainable Development Goals (SDGs) [5]. Maternal mortality is preventable and represents a violation of fundamental human rights, reflecting health and socioeconomic inequalities. Brazil has implemented policies to reduce maternal mortality since the 1980s, with a decline from 1990 to 1999, stable mortality from 2000 to 2013, and a new decline from 2017 to 2019 [6, 7]. Despite these historical trends, Brazil has not met its MDG targets and is not on track to meet its SDG targets by 2030 [5]. Recent studies link the mismanagement of the COVID-19 crisis to the reversal of declining trends in maternal mortality [8].

Identifying a typical profile associated with maternal mortality risk in Brazil, controlling for variables such as age and education, reveals inequalities. However, Brazil's continental size and diverse realities affect women's health unevenly. It is crucial to address regional inequalities, taking into account sociodemographic specificities, historical context, and cultural nuances [6]. The study of specific factors associated with obstetric causes is essential, as it highlights the importance of maternal and child health indicators. These indicators reflect the quality of obstetric care in Brazil. Addressing this issue comprehensively, considering individual characteristics, the regional context and the Unified Health System (SUS), can help develop more effective strategies to promote maternal health and reduce maternal mortality in the country.

Given this, the alternative hypothesis for the empirical analysis in this study is that sociodemographic factors and maternal-child health indicators predict maternal mortality from obstetric causes, both directly and indirectly, in Brazil. This study aimed to analyze maternal mortality from obstetric causes in Brazil from 2011 to 2021. To achieve this goal, 1) we examined the influence of sociodemographic factors in predicting obstetric mortality during pregnancy, childbirth, and the postpartum period in Brazil from 2011 to 2021; 2) we verified the influence of sociodemographic factors in predicting obstetric mortality (direct and indirect); and 3) we estimated whether sociodemographic variables and maternal and child health indicators predict the specific risk of maternal mortality from obstetric causes in Brazil from 2011 to 2021.

## Methods

### Design, area, population, and study period

**Fig. 1:**
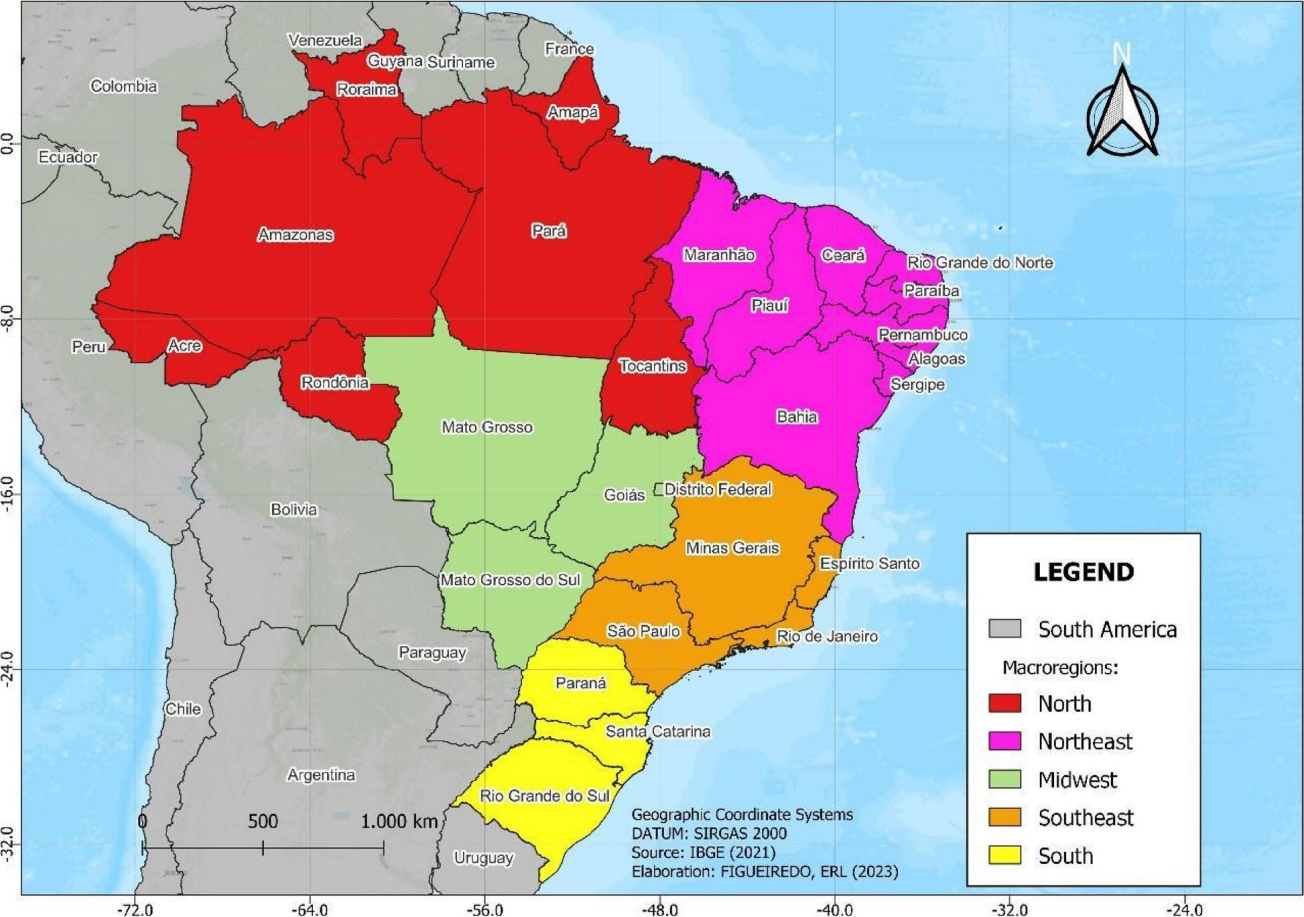
States and geographic regions of Brazil

An observational ecological study with descriptive and inferential analysis [9] was conducted in Brazil according to macroregion (North, Northeast, Midwest, Southeast and South) (Fig. 1) according to the current demarcation of the Political-Administrative Division (DPA), which covers an area of 8,510,345.540 square kilometers and is composed of 5,570 municipalities in 27 federal units (UF), 26 of which are states and one is a federal district [10].

The population and study period were composed of women reproductive-aged (WRA) between 10 and 49 years who died maternally due to obstetric causes (direct and indirect) between 2011 and 2021 according to the municipality of residence, federal unit and geographic region. The survey from 2011 was due to the beginning of the consolidation and standardization of the new forms of the death certificate (DO) and the Declaration of Live Birth (DN), with data available for free access on the platform of the Department of Informatics of the Unified Health System of Brazil (DATASUS), and ended in 2021, since it was the maximum period with data availability [11]. It is worth noting that there has been progress in the coverage and quality of data on the causes of WRA deaths [11].

### Data collection and sources

The data were obtained from the DO and DN databases of the systems managed by the DATASUS open access platform [11] of the Mortality Information System (MIS) and Live Birth Information System (LBIS) using the Tabwin data transfer application. The microdatasus package was also used by the RStudio team [12] (RStudio: Integrated Development Environment for R) to acquire the dbf database and preprocess and tabulate the microdata files in dbf format. Both systems are important tools for the development of more effective public health and social security policies.

The spatial data for the thematic presentation were obtained from the website of the Brazilian Institute of Geography and Statistics (IBGE) from the Digital Municipal Grids of the Political-Administrative Division of Brazil, according to the current political-administrative structure. The cartographic bases provided are appropriate for the original scale of 1:250,000 used in the project and use the Geodetic Reference System (SIRGAS 2000) in conjunction with the Geographic Coordinate System and UTF-8 text encoding. The data were processed with QGIS Desktop version 3.26.

### Eligibility criteria, operational procedures, and variables

**Fig. 2:**
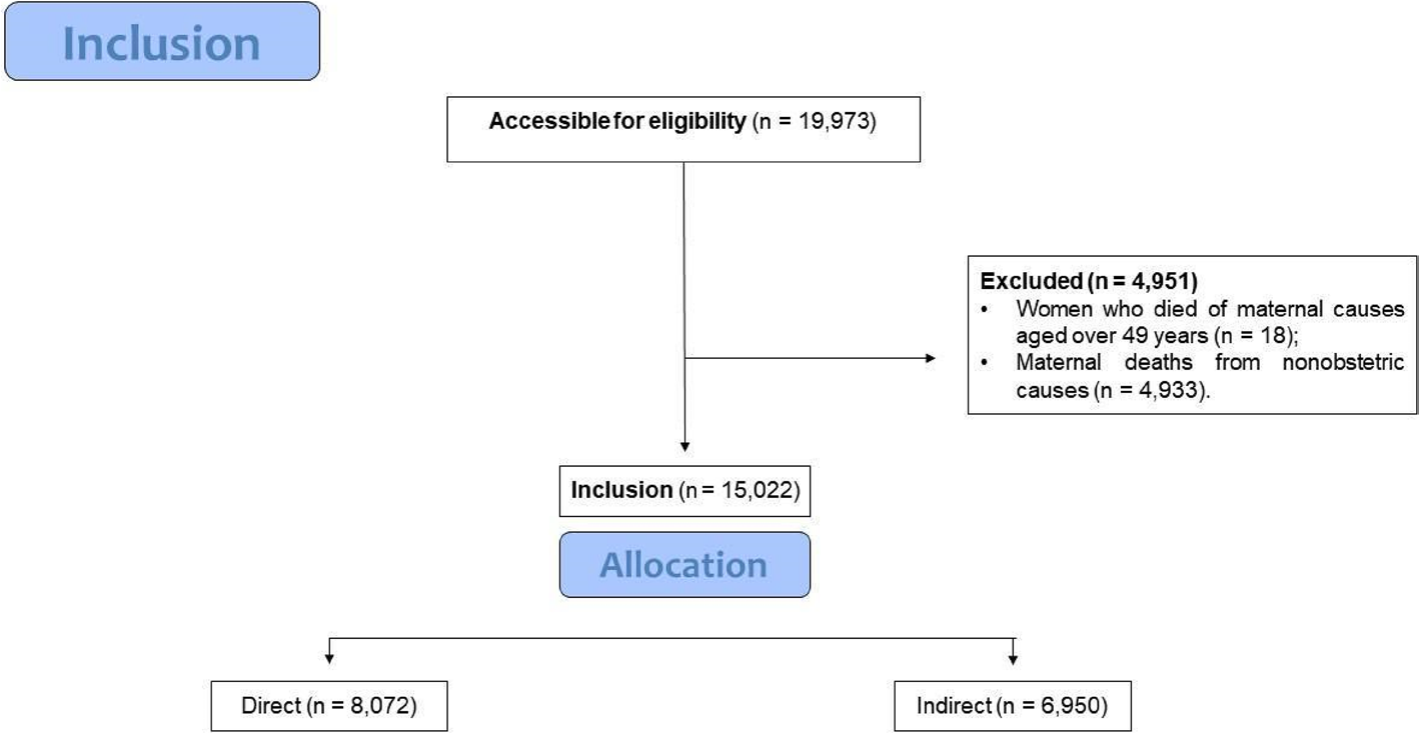
Data eligibility flow diagram

The deaths of WRA according to the place of residence of the deceased in the period from 2011 to 2021 were included, according to the type of obstetric cause, direct (coded in the ICD-10 as O00.0 to O08.9, O11 to O23.9, O24.4, O26.0 to O92.7, D39.2, E23.0) and indirect (coded in the ICD-10 as O10.0 to O10.9; O24.0 to O24.3; O24.9, O25, O98.0 to O99.8, A34, B20 to B24). Deaths of women older than 49 years of age and deaths in which the underlying cause did not include ICD-10 obstetric cause values were excluded. Figure 2 shows the eligibility criteria for the study population for testing the study hypotheses.

Once the data had been tabulated in the RStudio environment, the exploratory data surveillance analysis stages began to construct dependent and independent variables. A classification for understanding the variables studied is presented in Table 1.

**Table 1:** Classification of variables

Variable	Description	Source
*Dependent*		
Type of Obstetric Death	A dichotomous variable that classifies the occurrence of obstetric death into two different categories: "Direct" indicates the presence of direct obstetric death, while "Indirect" indicates indirect obstetric death	MIS
Death During Pregnancy	Dichotomous variable classifying whether or not the death occurred during pregnancy. A response of "Yes" indicates that the death occurred during pregnancy; a response of "No" indicates that the death occurred outside of pregnancy	MIS
Death in Puerperium	Dichotomous variable classifying whether or not death occurred in the postpartum period. A response of "Yes" indicates that the death occurred in the immediate postpartum period, which includes up to 42 days after birth. A response of "No" indicates that the death occurred outside this period	MIS
Specific Mortality Risk	Quantitative measures adjusted for year, certain sociodemographic characteristics, including age group, race/ethnicity, education, marital status, and method of death investigation. This adjustment allows for a more refined analysis that accounts for differences in the demographic composition of the study population	MIS and LBIS
*Independent*		
Year	Each year from 2011 to 2021, obstetric deaths and specific mortality risk are adjusted	MIS and LBIS
Place of residence	Obstetric deaths and specific mortality risk are adjusted for place of residence (federal unit, state, and city of the reproductive-age woman)	MIS and LBIS
Age Group	Age group of women of childbearing age according to the Brazilian classification, 10 to 14 years, 15 to 19 years, 20 to 29 years, 30 to 39 years, and 40 to 49 years	MIS
Race/Ethnicity	Refers to the classification of the population according to characteristics related to ethnic and racial origin. The categories used in Brazil are: white, black, brown, yellow, and indigenous	MIS
Schooling	It refers to the level of formal education an individual has attained. Categories include: no education, 1 to 3 years of study, 4 to 7 years, 8 to 11 years, and 12 or more years of study	MIS
Marital Status	Refers to the legal or social status of women of childbearing age in terms of marital relationships. Categorized as single, married, divorced, widow, and stable union	MIS
Source of Death Investigation	Refers to the source of information used to investigate the circumstances and causes of death. The categories included are: Home Visit (Family), Medical Records Forensic, Medical Institute, and Death Verification Service	MIS
Proportion of cesarean births	Refers to the percentage or share of total births that are by cesarean section (C-section) rather than vaginal delivery	LBIS
Proporcion of live births to mothers who had 3 or fewer antenatal visits	Is a health indicator that assesses the percentage of live births where the mothers had a relatively low number of antenatal (prenatal) visits during pregnancy	LBIS
Number of live births to adolescent mothers (Age group 10 to 19 years)	The average number of live births borne by a woman, by a specific age group of the reproductive period, in the total population residing in a given geographical area, in the year under consideration	LBIS

*LBIS* Live Birth Information System, *MIS* Mortality Information System

The specific mortality risk (SMR) was calculated for Brazil, region, and federative unit and adjusted for WRA characteristics according to age group (10 to 14 years, 15 to 19 years, 20 to 29 years, 30 to 39 years and 40 to 49 years), race/ethnicity (white, black, yellow, brown and indigenous), schooling (no years of study, 1 to 3 years, 4 to 7 years, 8 to 11 years and 12 years and over) and marital status (single, married, widowed, legally separated and cohabiting). Unlike studies that use adjusted mortality ratios [13–15] to investigate mortality, the adjusted SMR is a measure used to assess the likelihood of a woman dying from obstetric factors compared to a similar reference population, taking into account the variables studied [2, 3]. The other dependent variables in the study were categorical and dichotomous and included the type of obstetric death (direct or indirect), death during pregnancy (yes or no), or death during the puerperium (yes, within 42 days of delivery or no). It was not possible to obtain this variable in the delivery period because the corresponding values mostly indicated zero data.

The independent variables are the sociodemographic characteristics mentioned above for the SRM calculations, grouped into categories, and the source of death investigated [Maternal Mortality Committee, Home Visit (Family), Medical Record, Medical-Legal Institute, and Death Verification Service]. The other factors are continuous variables calculated from the LBIS information: the proportion of cesarean births out of the total number of births (vaginal or cesarean) by year and place of residence of the mother multiplied by 100; the proportion of live births to mothers who had 3 or fewer antenatal visits divided by the live births to mothers who had 4 or more antenatal visits multiplied by 100; and the number of live births to adolescent mothers (age groups 10 to 19 years) per 1,000 women in these age groups. Each of these variables provides specific and relevant information for the analysis of maternal mortality risk in the context of the study. The proportion of live births with mothers who had an inadequate number of visits (3 or fewer) compared with those who had a more complete follow-up (4 or more) is a sensitive measure for assessing access to and quality of prenatal care, and the number of live births to adolescent mothers is a measure of the prevalence of adolescent pregnancy, which allows us to assess how this specific population may contribute to the risk of maternal mortality from direct obstetric causes.

### Statistical analysis

Descriptive statistics were expressed using measures of central tendency, with the mean for parametric data and median for nonparametric data, and measures of dispersion [standard deviation (parametric) or minimum, maximum, and interquartile range (nonparametric)]. The absolute values of maternal deaths from obstetric causes (direct and indirect) and live births by year of birth and by mother's residence (region and UF) were used to construct direct and indirect SRMs adjusted for WRA characteristics of age group, race/ethnicity, marital status, and education. The data were subsequently tabulated in SPSS software version 27.0 (*IBM SPSS Statistics for Windows*, Version 27.0). The descriptive and inferential statistics were obtained from Armonk, NY, IBM Corp.

Binary logistic regression was used to 1) assess the influence of sociodemographic factors in predicting obstetric mortality (directly and indirectly) and 2) examine the influence of sociodemographic variables on the time of death (during pregnancy and postpartum). We checked the necessary assumptions to ensure the validity and correct interpretation of the results [16]. First, collinearity between the independent variables was diagnosed using the variance inflation factor (VIF), with the criterion that it should have a value less than 10 and a tolerance of less than 0.2. The "backward: conditional" method in SPSS was used to select the variables for the model. This iterative method adds and removes variables from the model, evaluating the quality of the fit at each step. The decision to add or remove variables was based on statistical criteria, such as a *p* value < 0.05, which indicates acceptance of the hypothesis that the model with the predictor variables provides a significantly different fit than the model without the predictor variables. The quality of the model fit was assessed using log likelihood. We also calculated Nagelkerke's R^2^, a measure of fit that ranges from 0 to 1 and indicates the proportion of total variation in the dependent variable that is explained by the model. Nagelkerke's R^2^ is an adjusted version of Cox and Snell's R^2^, calculated as the ratio of the log-likelihood of the full model to the log-likelihood of the null model. The reference classification for categorical variables was based on the first category for each disposition according to the coding of the data values in the MIS and LBIS databases. Finally, we calculated the confidence intervals for the odds ratios with a significance level α set at 0.05.

Multiple linear regression was used to analyze whether the proportion of cesarean deliveries, the proportion of live births to mothers with 3 or fewer prenatal visits, the number of live births to teen mothers, and the region of WRA residence were able to predict SRM for each adjusted sociodemographic category (age group, race/ethnicity, education, and marital status).

To construct the SRM models for each sociodemographic group, the necessary assumptions for multiple linear regression were met [17]. First, the significance of each model was assessed by evaluating the calculated F-statistic and performing a hypothesis test. The null hypothesis that all regression coefficients (β) are statistically equal to zero (indicating the absence of a regression model) was rejected, indicating the presence of a significant regression model (*p* < 0.05). We then performed Durbin–Watson analysis to test for autocorrelation in the residuals of the model. The results were within the acceptable range of 1.5 to 2.5, indicating independence of the residuals. Collinearity was diagnosed based on tolerance (values greater than 0.2) and VIF (values less than 10), ensuring that the independent variables were not highly correlated. The absence of outliers was confirmed by analyzing the standardized residuals, with reference values between -3 and +3. Finally, we evaluated the interdependence between the residuals and Cook's distance for each observation. The reference value for the interdependence between the residuals was between 1.5 and 2.5, and for Cook's distance, it was less than 1.

## Results

The number of live births in Brazil from 2011 to 2021 was 31,702,562, while the number of maternal deaths from obstetric causes studied during the same period was 15,022, 8,072 of which were attributed to direct causes and 6,950 to indirect causes. In terms of data completeness, for the race/ethnicity variable, approximately 3.42% of the data for direct obstetric causes were not correctly completed, while for the indirect causes dataset, this figure is slightly lower at approximately 3.06%. For marital status, 8.13% for direct causes and 7.53% for indirect causes were not filled in correctly or were ignored. The education variable has the highest percentage of uncorrected data, with 15.35% for direct causes and 14.59% for indirect causes.

### Sociodemographic predictors of pregnancy, delivery and postpartum mortality

First, the prediction of total obstetric mortality during pregnancy, childbirth, and the postpartum period was analyzed. As shown in Table 2, a binary logistic regression model was used to test whether sociodemographic factors were predictors of obstetric death during pregnancy, and a significant model was selected [χ^2^ (25) = 165.943; *p* < 0.001; R^2^ Nagelkerke = 0.044]. The odds of obstetric mortality during pregnancy increased over the years (OR: 1.029; 95% CI: 1.009–1.048; *p* = 0.003). Residence in the northern region was associated with lower odds of mortality during pregnancy than was residence in the central-west (OR: 1.431; 95% CI: 1.085–1.888; *p* = 0.011), south (OR: 1.414; 95% CI: 1.067–1.874; *p* = 0.016), or northeast (OR: 1.368, 95% CI: 1.082–1.730; *p* = 0.009) regions. The Maternal Mortality Committee was the primary source of investigation for obstetric deaths during pregnancy compared to others: Institute of Forensic Medicine (OR: 0.231, 95% CI: 0.158–0.339, *p* < 0.0001); Death Verification Service (OR: 0.309, 95% CI: 0.203–0.470, *p* < 0.0001); Home Visit (Family) (OR: 0.587, 95% CI: 0.466–0.739, *p* < 0.0001); and Medical Record (OR: 0.717, 95% CI: 0.615–0.837, *p* < 0.0001).

**Table 2:** Relationships between sociodemographic factors, source of investigation and obstetric deaths during pregnancy

				**Collinearity Statistics**	
	**ODDS RATIO**	**CI 95%**	***p* value**	**Tolerance**	**VIF**
**Year**	1.029	(1.009–1.048)	0.003	0.963	1.038
**Region (North)**			0.038	0.888	1.126
Northeast	1.368	(1.082–1.730)	0.009		
Southeast	1.251	(0.992–1.578)	0.059		
South	1.414	(1.067–1.874)	0.016		
Midwest	1.431	(1.085–1.888)	0.011		
**Age group (10 to 14 years)**			0.551	0.962	1.040
15 to 19 years	1.313	(0.632–2.729)	0.465		
20 to 29 years	1.389	(0.678–2.847)	0.369		
30 to 39 years	1.420	(0.691–2.916)	0.340		
40 to 49 years	1.625	(0.771–3.426)	0.202		
**Race/Ethnicity (White)**			0.906	0.907	1.103
Black	1.101	(0.897–1.351)	0.358		
Yellow	1.039	(0.332–3.254)	0.948		
Brown	1.000	(0.867–1.154)	1.000		
Indigenous	1.030	(0.554–1.916)	0.925		
**Marital status (Single)**			0.663	0.974	1.027
Married	1.099	(0.949–1.272)	0.207		
Widowed	1.473	(0.632–3.436)	0.370		
Separated	1.208	(0.678–1.560)	0.896		
Common-law marriage	1.000	(0.832–1.202)	0.997		
**Education (None)**			0.154	0.919	1.088
1 to 3 years	0.856	(0.520–1.412)	0.543		
4 to 7 years	1.078	(0.666–1.746)	0.759		
8 to 11 years	1.084	(0,672–1.729)	0.741		
12 or more years	1.175	(0.711–1.943)	0.529		
**Source of investigation (Maternal Mortality Committee)**			< 0.001	0.930	1.064
Home Visit (Family)	0.587	(0.466–0.739)	< 0.001		
Medical Records	0.717	(0.615–0.837)	< 0.001		
Forensic Medical Institute	0.231	(0.158–0.339)	< 0.001		
Death Verification Service	0.309	(0.203–0.470)	< 0.001		

*p* < 0.05, binary logistic regression with main effects

The prediction of obstetric mortality during childbirth was not feasible because 91.5% of the cases were omitted and there were no records of deaths during childbirth in Brazil. Subsequently, the significant model was selected [χ^2^ (25) = 184.501; *p* < 0.001, R^2^ Nagelkerke = 0.049] to identify sociodemographic variables predicting mortality in the puerperium up to 42 days after birth (Table 3). The odds of obstetric mortality at the puerperium decreased over the years (OR = 0.973, 95% CI = 0.955–0.991, *p* = 0.004). Residence in the northern region was more strongly associated with puerperium mortality than was residence in the mid-western region (OR = 0.741, 95% CI = 0.561–0.979, *p* = 0.035). The Maternal Mortality Committee was the least active source of investigation in the puerperium compared to the Forensic Medical Institute (OR: 4.681, 95% CI: 3.145–6.970, *p* < 0. 0001), death verification service (OR: 3.192, 95% CI: 2.082–4.892, *p* < 0.0001), home visit (family) (OR: 1.867, 95% CI: 1.482–2.352, *p* < 0.0001), and medical records (OR: 1.485, 95% CI: 1.274–1.732, *p* < 0.0001).

**Table 3:** Relationships between sociodemographic factors and sources of investigation and obstetric deaths in the puerperium

				**Collinearity Statistics**	
	**Odds ratio**	**CI 95%**	***p* value**	**Tolerance**	**VIF**
**Year**	0.973	(0.955–0.991)	0.004	0.930	1.038
**Region (North)**			0.280	0.885	1.130
Northeast	0.824	(0.652–1.041)	0.115		
Southeast	0.852	(0.675–1.075)	0.177		
South	0.767	(0.580–1.015)	0.063		
Midwest	0.741	(0.561–0.979)	0.035		
**Age group (10 to 14 years)**			0.294	0.962	1.039
15 to 19 years	0.646	(0.305–1.370)	0.255		
20 to 29 years	0.587	(0.280–1.227)	0.157		
30 to 39 years	0.566	(0.270–1.187)	0.132		
40 to 49 years	0.507	(0.236–1.088)	0.081		
**Race/Ethnicity (White)**			0.855	0.904	1.106
Black	0.931	(0.759–1.142)	0.491		
Yellow	1.225	(0.420–3.568)	0.710		
Brown	1.031	(0.894–1.190)	0.673		
Indigenous	0.907	(0.486–1.694)	0.759		
**Marital status (Single)**			0.846	0.974	1.027
Married	0.940	(0.812–1.089)	0.409		
Widowed	0.737	(0.328–1.661)	0.461		
Separated	1.068	(0.708–1.609)	0.755		
Common-law marriage	0.995	(0.828–1.195)	0.956		
**Education (None)**			0.103	0.917	1.090
1 to 3 years	1.176	(0.710–1.947)	0.529		
4 to 7 years	0.953	(0.586–1.549)	0.845		
8 to 11 years	0.930	(0.574–1.507)	0.768		
12 or more years	0.821	(0.495–1.363)	0.446		
**Source of investigation (Maternal Mortality Committee)**			< 0.001	0.928	1.077
Home Visit (Family)	1.867	(1.482–2.352)	< 0.001		
Medical Records	1.485	(1.274–1.732)	< 0.001		
Forensic Medical Institute	4.681	(3.145–6.970)	< 0.001		
Death Verification Service	3.192	(2.082–4.892)	< 0.001		

*p* < 0.05, binary logistic regression with main effects

### Sociodemographic predictors of mortality from direct vs. indirect obstetric causes

Subsequently, sociodemographic factors predicting death from obstetric causes, examining the differences between direct and indirect causes, were identified by binary logistic regression (Table 4), with a significant model selected [χ^2^ (25) = 287.285; *p* < 0.001; R^2^ Nagelkerke = 0.068]. The odds of death from indirect obstetric causes increased with age (OR: 1.126; 95% CI: 1.106–1.145; *p* < 0.001). The northern region had greater odds of death from direct obstetric causes than did the southeastern region (OR: 1.336; 95% CI: 1.068–1.670; *p* = 0.011). Regarding race/ethnicity, compared with white ethnicity, indigenous ethnicity (OR: 0.502; 95% CI: 0.264–0.955; *p* = 0.036), black ethnicity (OR: 0.764; 95% CI: 0.636–0.918, *p* = 0.004), and brown ethnicity (OR: 0.823; 95% CI: 0.723–0.937; *p* = 0.003) were more strongly associated with mortality from direct causes. Regarding marital status, being in a stable union was associated with a lower odds of mortality from indirect obstetric causes than being single (OR = 0.764; 95% CI = 0.642–0.904; *p* = 0.002).

**Table 4:** Relationship of sociodemographic factors with mortality from obstetric causes: Differences between direct and indirect causes

				**Collinearity Statistics**	
	**Odds ratio**	**CI 95%**	***p* value**	**Tolerance**	**VIF**
**Year**	1.126	(1,106–1,145)	< 0.001	0.965	1.036
**Region (North)**			0.005	0.889	1.125
Northeast	1.000	(0.786–1.236)	0.903		
Southeast	1.336	(1.068–1,670)	0.011		
South	1.120	(0,860–1.459)	0.399		
Midwest	1.198	(0.945–1.592)	0.125		
**Age group (10 to 14 years)**			< 0.001	0.962	1.039
15 to 19 years	1.401	(0.662–2.966)	0.378		
20 to 29 years	2.064	(0.988–4.314)	0.054		
30 to 39 years	1.785	(0.853–3.737)	0.124		
40 to 49 years	1.743	(0.817–3.720)	0.151		
**Race/Ethnicity (White)**			0.005	0.907	1.103
Black	0.764	(0.636–0.918)	0.004		
Yellow	0.650	(0.234–1.806)	0.409		
Brown	0.823	(0.723–0.937)	0.003		
Indigenous	0.502	(0.264–0.955)	0.036		
**Marital status (Single)**			0.024	0.975	1.026
Married	0.890	(0.779–1.014)	0.079		
Widowed	0.958	(0.453–1.811)	0.780		
Separated	0.786	(0.546–1.144)	0.213		
Common-law marriage	0.764	(0.642–0.904)	0.002		
**Education (None)**			0.523	0.922	1.084
1 to 3 years	1.117	(0.690–1.806)	0.653		
4 to 7 years	1.042	(0.656–1.655)	0.861		
8 to 11 years	1.041	(0.658–1.648)	0.864		
12 or more years	1.198	(0.743–1.933)	0.458		
**Source of investigation (Maternal Mortality Committee)**			0.373	0.931	1.074
Home Visit (Family)	1.041	(0.837–1.295)	0.718		
Medical Records	0.892	(0.775–1.026)	0.109		
Forensic Medical Institute	0.869	(0.602–1.254)	0.454		
Death Verification Service	1.000	(0.570–1.292)	0.465		

*p* < 0.05, binary logistic regression with main effects

Multiple linear regression analysis was subsequently performed to identify sociodemographic variables and maternal and child health indicators that directly or indirectly predicted the SMR. The results of the multiple linear regression analyses for each adjusted SMR are presented in Table 5.

**Table 5:** Impact of maternal-child indicators on specific risk of mortality (SMR) from direct and indirect obstetric causes

				**Collinearity Statistics**	
	**Standardization Coefficient β**	**t**	***p* value**	**Tolerance**	**VIF**
**SRM Direct**					
***Age Group***					
**15 to 19 years**					
Cesarean Deliveries	-0.420	-3.710	< 0.001	0.675	1.480
Live births from women who had 3 or fewer prenatal visits	0.416	3.673	< 0.001	0.675	1.480
***Education***					
**8 to 11 years**					
Live births from women who had 3 or fewer prenatal visits	0.452	2.433	0.018	0.385	2.599
***Marital Status***					
**Single**					
Live births from women who had 3 or fewer prenatal visits	0.756	4.782	< 0.001	0.343	2.916
**Married**					
Cesarean Deliveries	-0.384	-2.660	0.010	0.452	2.211
Live births from women who had 3 or fewer prenatal visits	0.408	2.717	0.009	0.416	2.402
**Common-law Marriage**					
Live births from women who had 3 or fewer prenatal visits	0.872	3.891	< 0.001	0.207	4.823
Live births from adolescent mothers (age groups 10–14 and 15–19)	-0.407	-3.737	< 0.001	0.876	1.141
**SRM Indirect**					
***Education***					
**No years of study**					
Cesarean Deliveries	0.803	2.132	0.038	0.108	9.235
***Marital Status***					
**Divorced**					
Cesarean Deliveries	0.601	2.604	0.012	0.307	3.352
Region	-0.644	-2.779	0.008	0.304	3.285

*p* < 0.05, multivariate linear regression analysis

### Sociodemographic factors and maternal and child health indicators as predictors of direct SMR

For the direct SMR by age group, the model was significant [F(2,52) = 31.667; *p* < 0.001; *R* = 0.741; R^2^ = 0.549]. For women aged 15–19 years, an increase in the proportion of cesarean deliveries reduced the risk of death (β = -0.420; t = -3.710; *p* < 0.001). In addition, mortality was greater among women who had 3 or fewer antenatal visits (beta = 0.416; t = 3.673; *p* < 0.001).

For the direct SMR by education, the model [F(2,52) = 11.958; *p* < 0.001; *R* = 0.561; R^2^ = 0.315] indicated that women who completed high school (8 to 11 years of education) had higher mortality when they had fewer than 4 prenatal visits (β = 0.452; t = 2.433; *p* = 0.018).

For the direct SMR by marital status, the model [F(3,51) = 21.853; *p* < 0.001; *R* = 0.750; R^2^ = 0.562] indicated that unmarried women had higher mortality when they had fewer than 4 antenatal visits (β = 0.756; t = 4.782; *p* < 0.001). In addition, the proportion of cesarean births (β = -0.384; t = -2.660; *p* = 0.010) and the proportion of live births among women who had 3 or fewer antenatal visits (β = 0.408; t = 2.717; *p* = 0.009) are predictors of direct SMR in married WRAs [F(3,51) = 18.415; *p* < 0.001; *R* = 0.721; R^2^ = 0.520]. For stable union, the model [F(3,51) = 15.017; *p* < 0.001; *R* = 0.685; R^2^ = 0.469] indicated that the proportion of live births to women who had 3 or fewer prenatal visits (β = 0.872; t = 3.891; *p* < 0.001) and the number of live births to teenage mothers (β = -0.407; t = -3.737; *p* < 0.001) were predictors.

### Sociodemographic variables and maternal and child health indicators predicting indirect SMR

Women with no education [F(2,52) = 6.558; *p* = 0.003; *R* = 0.449; R^2^ = 0.201], who had more cesarean deliveries (β = 0.803; t = 2.132; *p* < 0.038), and who were divorced [F(4,50) = 2.784; *p* = 0.036; *R* = 0. 425; R^2^ = 0.182], who had more cesarean deliveries (β = 0.601; t = 2.604; *p* < 0.012), and who lived in the north and northeast (β = -0.644; t = -2.779; *p* < 0.008) had higher indirect SMRs.

## Discussion

Based on the conclusions of this study, we can confirm this alternative hypothesis since significant associations were found between sociodemographic factors and maternal-infant health indicators and between maternal mortality from obstetric causes, both direct and indirect, in the Brazilian context. This highlights the need for epidemiologic surveillance of adjusted maternal mortality, either through the SMR or the adjusted morbidity rate [2, 3, 13–15].

In this study, an increase in obstetric mortality during pregnancy was observed over the years. A previous study highlighted that maternal mortality in Brazil is considered very high, and the country faces several challenges [18], in addition to considering the impact of COVID-19 [19, 20].

Furthermore, although analysis of the temporal trend of increasing obstetric deaths during childbirth over the years has been hampered by incomplete data, the literature points to gaps in care and the need to prevent more common complications, such as hemorrhage [21], hypertension [22], puerperal infection [23], labor mismanagement, uterine rupture, and anemia [24]. However, a decrease in postpartum deaths has been observed despite deficits in human and material resources, low coverage of postpartum and home visits, and a focus on care in the immediate postpartum period. Improvements in this condition in Brazil have been associated with strengthening the physical structure for postpartum care and screening for postpartum depression [25].

Another critical issue to address is the increase in mortality from indirect obstetric causes during the study period, a trend observed globally. To help countries get back on track in reducing preventable maternal deaths and to monitor progress toward sustainable development goals (SDGs), the World Health Organization (WHO) has set five key targets. Globally, maternal mortality decreased by more than one-third between 2000 and 2017 [23]. However, the COVID-19 pandemic has caused significant disruptions to health services, exacerbating these risks, especially for the most vulnerable families [18, 26].

Our findings also highlight regional disparities in the health of Brazilian women. The northern region had a greater probability of death from obstetric causes during the postpartum period and from direct obstetric causes. This may be related to the socioeconomic conditions and access to health services for women in this region, who face greater vulnerability and lower coverage of prenatal and institutional delivery services [25]. Residence in northern Brazil was associated with lower odds of mortality during pregnancy. Divorced women, especially in the North and Northeast regions, were more likely to die from indirect obstetric causes.

The northern region is also notable for encompassing the political-administrative region of the Brazilian Legal Amazon, which, in addition to its megabiodiversity, contains a great sociometabolic complexity that strongly influences the social and cultural aspects of this population [27]. These factors, combined with the globally observed inequalities in reproductive and child health, contribute to the impact of demographic disparities on maternal morbidity and mortality [28–30], which is particularly evident in the continental dimension of Brazil. In addition, the northern region was one of the regions most affected by the COVID-19 pandemic, which may have contributed to the increase in postpartum maternal mortality [31].

The findings also highlight the structural racism that affects the health of black and indigenous women, who have historically experienced violations of their reproductive rights and safe motherhood [32]. A higher likelihood of maternal mortality among black women has also been reported in other parts of the world, with studies in North America [33] and Latin America [34] highlighting that racial disparities have a significant impact on maternal mortality, with significantly higher rates among black women than white women. The increased likelihood of mortality from direct obstetric causes resulting from complications of pregnancy, childbirth, and the postpartum period and related to lack of access to and quality of maternal health care among black and indigenous women also highlights the need for increased efforts to address disparities in the SUS. It is important to note the recent pandemic scenario where the maternal mortality rate due to COVID-19 was almost double that of white women among black women [35].

Marital status, a predictor of mortality, also yielded significant results; in general, unmarried women had a greater risk than married or cohabiting women did. This result is consistent with other findings [36, 37]. However, marital status remains a relatively underdiscussed variable in terms of its impact on maternal morbidity and mortality. Several hypotheses may explain the increased vulnerability of unmarried women to specific obstetric mortality. One possibility is that unmarried women may have less access to and lower quality of health services, especially for antenatal care, childbirth and the postpartum period. This may be related to socioeconomic, cultural, and geographical factors that hinder proper and timely monitoring [38]. Another hypothesis is that unmarried women may have less social, financial, and emotional support from their partners, family, and community, which negatively affects their physical and mental health and their ability to cope with obstetric complications [39]. It is therefore essential to recognize marital status as a social determinant of maternal health and to develop intervention strategies that take into account the specificities and needs of unmarried women. Finally, unmarried, married, and cohabiting women with fewer than 4 antenatal care visits had an increased risk of death from direct obstetric causes.

Education, or years of schooling, has also been described in the literature as a determining and structuring factor in maternal mortality. For example, a study in Asia [40] revealed that educational inequality was an important factor in maternal mortality, with significantly greater rates of education among women with lower education levels. In our study, inadequate antenatal care was a predictor of the risk of direct obstetric mortality among women aged 15–19 years and those who completed high school. Cesarean delivery was a predictor of the risk of indirect obstetric mortality among divorced women and women with no years of education but was a protective factor among married women and women aged 15–19 years.

Several suggestions can be made to explain this association, including the possibility that women with low education levels may have less knowledge about the signs and symptoms of obstetric complications, as well as the necessary care during the prenatal, delivery, and postpartum periods. In addition, women with low levels of education may have less ability to communicate and negotiate with healthcare providers, making it difficult to establish a relationship of trust and mutual respect [41, 42].

The impact of maternal and child health indicators on obstetric mortality is also a critical issue that deserves further study. The number of antenatal visits stands out as an indicator of access to and quality of health services, as well as prevention and early detection of obstetric complications. Studies show that a low number of antenatal visits is associated with an increased risk of mortality from direct and indirect obstetric causes in Brazil and other countries [43, 44]. Prenatal care plays a critical role in the early detection of potential complications during pregnancy. Through regular visits, healthcare providers have the opportunity to monitor a pregnant woman's health, screen for risk factors, and implement preventive measures. However, when visits are infrequent or inadequate, these opportunities are missed, leaving women in a vulnerable position [45].

The influence of the cesarean delivery rate on maternal mortality from obstetric causes in Brazil is complex. The results of this study, based on multiple linear regression analysis, showed that cesarean delivery affects different groups of women of childbearing age. Among women aged 15–19 years, cesarean delivery was identified as a protective factor against SRM due to direct obstetric causes. This finding suggests that in early pregnancy, cesarean delivery may be beneficial for reducing direct obstetric complications that threaten the life of the mother [46]. This is an important finding given the frequency of adolescent pregnancies in Brazil. Among married women, cesarean delivery was also found to be a protective factor against mortality from direct obstetric causes, which may be related to easier access to health services and better childbirth preparation among married women, resulting in a lower incidence of severe complications [47]. However, the analysis also revealed a different scenario for the other groups; among divorced women and those with no formal education, cesarean delivery was associated with an increased risk of death from indirect obstetric causes. This raises important concerns about the practice of cesarean delivery among vulnerable women and its indiscriminate use [48].

For the indicators of live births to adolescent mothers and SRM due to direct causes among women in a stable union, we found that having live births at a young age may act as a protective factor against direct obstetric complications among women in a stable union. These findings highlight the complexity of the relationship between maternal age or marital status and maternal mortality. While early motherhood may be considered a risk factor in some circumstances, this study suggested that early motherhood may confer protection against direct obstetric complications when combined with a stable union. It is important to note that these findings should be interpreted with caution given the specific context of the study design [9], where not all situations fit this pattern and additional factors such as access to health care, quality of health services, and other social determinants of health play important roles. Thus, our results suggest that women who had children at younger ages and who maintained stable unions may have had greater opportunities to access maternal health care, education, and social support. It is important to emphasize that health policies should be developed on the basis of sound evidence and take into account the complex interactions between age, marital status and the risk of obstetric mortality. It is also essential to ensure equitable access to quality health care and maternal health education for all women, regardless of age and marital status [1].

### Strengths and limitations

The strengths of this study include the use of a comprehensive and reliable database that allows detailed analysis of maternal mortality from obstetric causes over the years. Advanced statistical techniques were used to analyze the relationships between a wide range of sociodemographic factors, including age group, race/ethnicity, region of residence, method of death investigation, marital status, and education level. This approach allowed for a more comprehensive analysis of the determinants of maternal mortality. Based on the findings of this study, maternal mortality from obstetric causes is a complex and multifaceted issue that requires a comprehensive approach. It is essential to consider the specificities of both direct and indirect causes, as well as the needs of women, considering the influence of sociodemographic factors and the quality of maternal and child health care.

It is important, however, to acknowledge their limitations. An inherent challenge of ecological studies is the possibility of committing an "ecological fallacy". This means that associations observed at the population level may not be true at the individual level. Therefore, conclusions about the relationship between sociodemographic variables and maternal mortality may not be directly generalizable to individual women. In addition, ecological studies do not allow for causal inference. The observed associations between sociodemographic variables and maternal mortality do not imply causality. Other confounding factors may affect these associations [49].

## Conclusion

This study revealed an increase in obstetric mortality during pregnancy over the years, with inconclusive information available for childbirth and a decreased likelihood of postpartum mortality. The northern region had lower odds of pregnancy-related mortality but higher odds of postpartum mortality than did the other regions. Divorced women, particularly those in the North and Northeast regions, had higher odds of indirect obstetric mortality. Indigenous, black, and brown women had higher odds of direct obstetric mortality than white women did. Stable union was protective against indirect obstetric mortality. Women with fewer than 4 antenatal visits had an increased risk of both direct and indirect obstetric mortality. Inadequate prenatal care predicted direct obstetric mortality among women aged 15–19 years and those with a high school education. Cesarean delivery was a predictor of indirect obstetric mortality in some groups but was a protective factor in others. The importance of studying causes of death, especially the role of the Maternal Mortality Committee, was emphasized. Sociodemographic factors and maternal and child health indicators showed different patterns of obstetric mortality.

### Supplementary Information

**Additional file 1.**

## Data Availability

The data used for the study are publicly available and can be accessed from the website (opendatasus.saude.gov.br) and Microdatasus in RStudio (check the script in the Supplementary material).

## Abbreviations

IBGE: Brazilian Institute of Geography and Statistics
DO: Death Certificate
DN: Declaration of Live Birth
DASNT: Department of Health Analysis and Surveillance of Non-Communicable Diseases
DATASUS: Department of Informatics of the Unified Health System of Brazil
UF: Federal units
LBIS: Live Birth Information System
MIS: Mortality Information System
SIRGAS 2000: Reference Geodetic System
SMR: Specific Mortality Risk
SDGs: Sustainable Development Goals
SUS: Unified Health System
VIF: Variance Inflation Factor
WRA: Women of reproductive-age
WHO: World Health Organization
